# Increase of Myocardial Ischemia Time and Short-Term Prognosis of Patients with Acute Myocardial Infarction during the First COVID-19 Pandemic Wave

**DOI:** 10.3390/medicina57121296

**Published:** 2021-11-25

**Authors:** Povilas Budrys, Mindaugas Lizaitis, Kamile Cerlinskaite-Bajore, Vilhelmas Bajoras, Greta Rodevic, Aurelija Martinonyte, Laurynas Dieckus, Ignas Badaras, Pranas Serpytis, Romualdas Gurevicius, Rasa Visinskiene, Romualdas Buivydas, Aleksandr Volodko, Egle Urbonaite, Jelena Celutkiene, Giedrius Davidavicius

**Affiliations:** 1 Clinic of Cardiac and Vascular Diseases, Faculty of Medicine, Vilnius University, 03101 Vilnius, Lithuania; kamile.cerlinskaite@santa.lt (K.C.-B.); vilhelmas.bajoras@santa.lt (V.B.); greta.rodevic@santa.lt (G.R.); aurelija.martinonyte@santa.lt (A.M.); pranas.serpytis@santa.lt (P.S.); jelena.celutkiene@santa.lt (J.C.); giedrius.davidavicius@santa.lt (G.D.); 2 Cardiology and Angiology Center, Vilnius University Hospital Santaros Klinikos, 08410 Vilnius, Lithuania; mindaugas.lizaitis@santa.lt (M.L.); egle.urbonaite@santa.lt (E.U.); 3 Faculty of Medicine, Vilnius University, 03101 Vilnius, Lithuania; laurynas.dieckus@gmail.com (L.D.); ignas.badaras@stud.mf.vu.lt (I.B.); 4 Center for Health Statistics, Institute of Hygiene, 01128 Vilnius, Lithuania; romualdas.gurevicius@hi.lt; 5 National Health Insurance Fund under the Ministry of Health, 09307 Vilnius, Lithuania; rasa.visinskiene@gmail.com; 6 Health Economics Centre, 10306 Vilnius, Lithuania; rbuivydas@sec.lt; 7 Emergency Medical Services Station, 05131 Vilnius, Lithuania; aleksandr.volodko@vgmps.lt

**Keywords:** COVID-19, myocardial infarction, ischemia time, percutaneous coronary intervention

## Abstract

*Background and objectives*: early reports showed a decrease in admission rates and an increase in mortality of patients with acute myocardial infarction (AMI) during the first wave of COVID-19 pandemic. We sought to investigate whether the COVID-19 pandemic and associated lockdown had an impact on the ischemia time and prognosis of patients suffering from AMI in the settings of low COVID-19 burden. *Materials and Methods:* we conducted a retrospective data analysis from a tertiary center in Lithuania of 818 patients with AMI. Data were collected from 1 March to 30 June in 2020 during the peri-lockdown period (2020 group; *n* = 278) and compared to the same period last year (2019 group; *n* = 326). The primary study endpoint was all-cause mortality during 3 months of follow-up. Secondary endpoints were heart failure severity (Killip class) on admission and ischemia time in patients with acute ST segment elevation myocardial infarction (STEMI). *Results:* there was a reduction of 14.7% in admission rate for acute myocardial infarction (AMI) during the peri-lockdown period. The 3-month mortality rate did not differ significantly (6.9% in 2020 vs. 10.5% in 2019, *p* = 0.341 for STEMI patients; 5.3% in 2020 vs. 2.6% in 2019, *p* = 0.374 for patients with acute myocardial infarction without ST segment elevation (NSTEMI)). More STEMI patients presented with Killip IV class in 2019 (13.5% vs. 5.5%, *p* = 0.043, respectively). There was an increase of door-to-PCI time (54.0 [42.0–86.0] in 2019; 63.5 [48.3–97.5] in 2020, *p* = 0.018) and first medical contact (FMC)-to-PCI time (101.0 [82.5–120.8] in 2019; 115 [97.0–154.5] in 2020, *p* = 0.01) during the pandemic period. *Conclusions:* There was a 14.7% reduction of admissions for AMI during the first wave of COVID-19. FMC-to-PCI time increased during the peri-lockdown period, however, it did not translate into worse survival during follow-up.

## 1. Introduction

The COVID-19 pandemic was associated with more than 4,000,000 deaths worldwide [1]. In Lithuania, the lockdown due to the first wave of COVID-19 was implemented on 16 March 2020 and lasted for three months. It was expected that high numbers of severely sick COVID-19 patients would overload hospitals, and therefore many elective health care services (including cardiac care) were halted and emergency care was reorganized.

To prevent the spread of infection in the hospital among medical staff and patients some precautionary measures were implemented like routine testing of patients for SARS-CoV-2 virus at the emergency ward, change in the treatment algorithms with emphasis to limit patient transfers between the hospitals, and usage of personal protective equipment (PPE) by medical workers. Inevitably, these changes caused delays in timely diagnosis and treatment of various diseases, and therefore possibly affected patient prognosis. On the other hand, movement inside the country was restricted, and media was crowded with information about the treatment of COVID-19 patients and the spread of coronavirus infection in hospitals among practitioners. In fact, little information was given on the awareness and readiness of hospitals to provide necessary cardiac care services.

All over Europe early reports showed a decrease in admission rates of patients with acute myocardial infarction [2,3,4,5,6,7,8,9,10], and in some countries there was a shift in myocardial infarction treatment options from percutaneous treatment to medical fibrinolysis [11].

We sought to investigate whether the COVID-19 pandemic and the first wave-related lockdown had an impact on the myocardial ischemia time, severity of presentation, and short-term prognosis of patients suffering from acute myocardial infarction in the tertiary Lithuanian center in the settings of low COVID-19 burden.

## 2. Methods

We conducted a retrospective data analysis from a single tertiary center in Lithuania (Vilnius University Hospital Santaros Klinikos), which serves a population of 1.2 million citizens. All patients from Vilnius and surrounding regions with acute myocardial infarction are referred for percutaneous coronary intervention (PCI) to this center. A 24/7 service was established here in 1995 with an annual load of primary PCIs of approximately 850.

Patients who presented with acute ST segment elevation myocardial infarction (STEMI) and acute myocardial infarction without ST segment elevation (NSTEMI) were included in the study.

Inclusion criteria:

- Diagnosis of STEMI and NSTEMI
- Coronary angiography performed
- Symptom onset time < 48 h for patients with STEMI

Exclusion criteria:

- Type 2 myocardial infarction
- Coronary angiography not performed
- Nonobstructive coronary artery disease
- STEMI or NSTEMI diagnosed while admitted to hospital for other reason
- Thrombolysis performed

**Figure 1:**
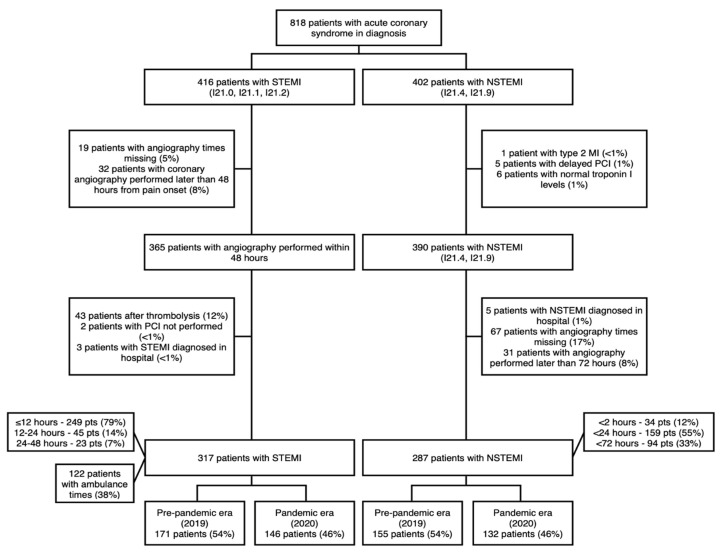
Clinical trial flow chart.

The flowchart of our trial is presented in Figure 1. 

The ambulance service notes (*n* = 122 were available) and hospital electronic case files (*n* = 818) were reviewed. 604 patients met inclusion and exclusion criteria and were included in the study. Official lockdown was announced on 16 March and canceled on 17 June 2020. Data were collected from 1 March to 30 June in 2020 (2020 group; *n* = 278) and compared to the same period last year (2019 group; *n* = 326). During the pandemic period, samples for polymerase chain reaction (PCR) SARS-CoV-2 test were taken from every patient at the time of arrival to our hospital and all patients were assumed COVID-19 positive until proven otherwise with PCR test. During coronary angiography and PCI procedures, cath-lab personnel used full PPE.

Demographic, clinical, and PCI procedure-related data were collected and compared between 2019 and 2020 groups. The timing of the chest pain onset, call for medical help, first medical contact (FMC), arrival to PCI center, and PCI procedure were analyzed.

The primary study endpoint was all-cause mortality during the hospital stay and at 3 months of follow-up. Secondary endpoints were heart failure severity (Killip class) on admission and ischemia time in patients with STEMI.

## 3. Statistical Analysis

Continuous variables are presented as median and interquartile ranges [IQR], categorical variables are presented as counts (percentage). The differences between study groups were compared using Kruskal–Wallis test for continuous variables and χ2 test for the categorical variables.

All statistical analyses were performed using R version 4.0.3 (The ‘R’ Foundation for Statistical Computing) with the statistical package “tableone” for statistical analysis. All tests were 2-sided and a p value of <0.05 was considered significant. No imputation was used for missing data.

This study complies with the Declaration of Helsinki, and the locally appointed ethics committee has approved the research protocol (number of approval Nr. 2020/8-1247-730).

## 4. Results

**Figure 2:**
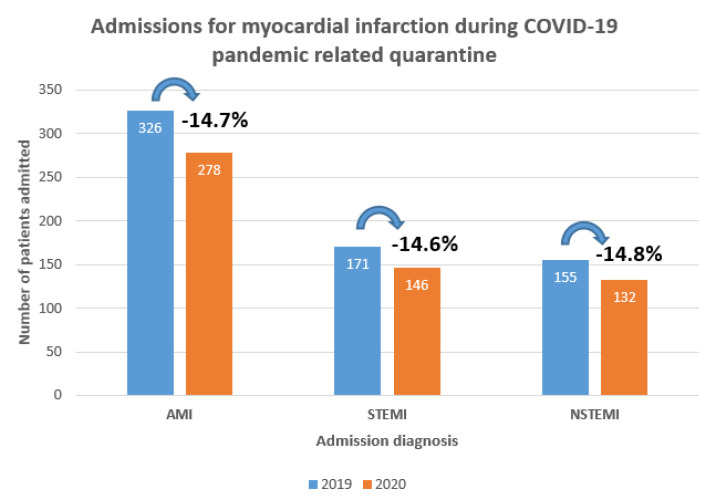
Admissions for myocardial infarction during COVID-19 related quarantine.

There was a reduction of 14.7% in admission rate for acute myocardial infarction (AMI) during the ‘peri-lockdown’ period in 2020 compared to 2019. Admission rates went down similarly for STEMI and NSTEMI patients (Figure 2). Baseline clinical characteristics of patients with AMI did not differ significantly between the two groups (Table 1). The median age was similar among STEMI (67.0 [59.0–76.5] in 2019, 65.0 [57.0–74.0] in 2020, *p* = 0.097) and NSTEMI patients (68.0 [59.0–78.0] in 2019, 68.5 [59.0–76.0] in 2020, *p* = 0.706). There were more patients with underlying arterial hypertension (84.8% in 2020 vs. 73.7% in 2019, *p* = 0.023) and dyslipidemia (89.7% in 2020 vs. 80.5% in 2019, *p* = 0.036) in 2020 among STEMI patients while the prevalence of these comorbidities did not differ significantly among NSTEMI patients. The frequency of type 2 diabetes mellitus in NSTEMI group and status of current smoking among STEMI patients were higher during peri-lockdown period.

The localization of AMI, rate of out-of-hospital cardiac arrest, peak high sensitivity Troponin I, left ventricle ejection fraction, and the length of hospital stay did not differ significantly between quarantine period and 2019 groups. However, more STEMI patients presented with Killip IV class in 2019 compared to that of 2020 (13.5% vs. 5.5%, *p* = 0.043, respectively), while there was no significant difference in Killip class among NSTEMI patients (Table 2).

Coronary angiography findings and procedural characteristics were similar in 2019 and 2020 groups (Table 3). The number of diseased vessels, distribution of culprit coronary artery, thrombolysis in myocardial infarction (TIMI) flow before and after PCI, complications related to PCI, the use of hemodynamic support devices did not differ between pandemic and pre-pandemic era. However, more NSTEMI patients were managed conservatively in 2020 (3.8%) compared to 2019 (0%), *p* = 0.033, while almost every STEMI patient underwent PCI.

Both STEMI and NSTEMI patients were more often referred to in-patient cardiac rehabilitation in 2019 (72.3% of STEMI patients and 57.6% of NSTEMI patients) in comparison to that of 2020 (56.7% of STEMI patients and 35.9% of NSTEMI patients), *p* < 0.01.

The adverse events of patients during hospital stay and 3 months follow-up are presented in Table 4. The frequency of worsening of heart failure, stroke and recurrent MI was similar between the patient groups during hospital stay. We did not find a statistically significant difference in the 3-month mortality rate in patients with AMI: 6.8% in 2020 vs. 10.5% in 2019, *p* = 0.341 for STEMI patients; 5.3% in 2020 vs. 2.6% in 2019, *p* = 0.374 for NSTEMI patients. Yet, in-hospital mortality of STEMI patients was higher in prepandemic era (9.4%) compared to that of 2020 (3.4%) group, and this difference narrowly missed statistical significance (*p* = 0.059).

**Figure 3:**
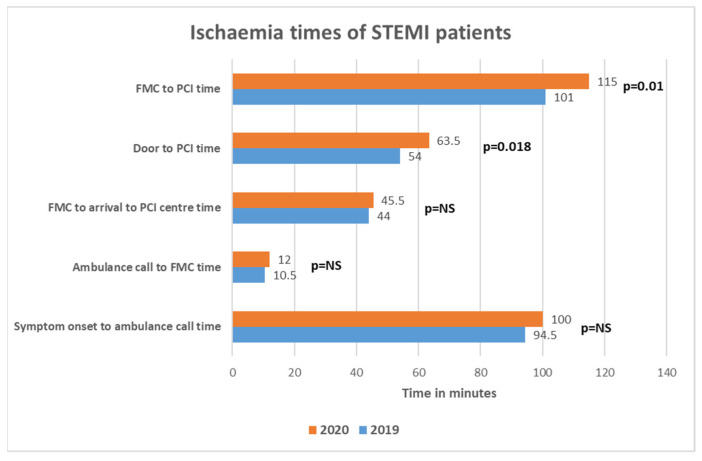
Ischemia times of patients presenting with STEMI.

**Figure 4:**
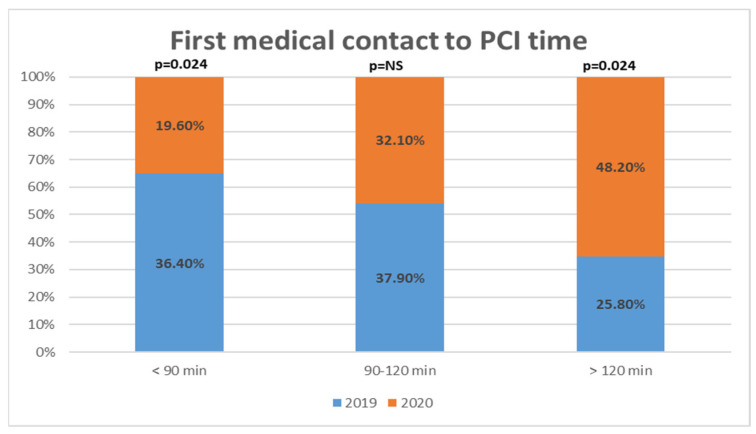
Patients presenting with STEMI distribution in different time intervals according to first medical contact to percutaneous coronary intervention time.

Myocardial ischemia times of patients admitted with STEMI are demonstrated in Figure 3 and Figure 4.

Time intervals from symptom onset to call for ambulance service, call for medical help to FMC and FMC to arrival to PCI center were similar between 2019 and 2020. There was a statistically significant increase of door to PCI time (54.0 [42.0–86.0] in 2019; 63.5 [48.3–97.5] in 2020, *p* = 0.018) and FMC to PCI time (101.0 [82.5–120.8] in 2019; 115 [97.0–154.5] in 2020, *p* = 0.01) during the peri-lockdown period. Almost half (48.2%) of STEMI patients had FMC to PCI time longer than ESC recommended 120 min in 2020 quarantine period, while there were only 25.8% of such patients in prepandemic era, *p* = 0.024.

## 5. Discussion

Our study overall confirmed and demonstrated that during the COVID-19 pandemic related lockdown: (1) the number of hospitalizations for acute myocardial infarction was reduced by 14.7% despite modest number of COVID-19 cases; (2) there was a delay in providing primary PCI service: time interval from the first medical contact to myocardial reperfusion was prolonged by 14 min, including 9 min delay from the hospital door to balloon; (3) however, all-cause mortality (in hospital and within 3 months after hospitalization) did not increase in our sample.

One of the possible explanations of decrease in acute MI admissions is that some patients suffering from myocardial infarction have chosen to stay at home, but not to seek for medical help due to the fear of getting infected by COVID-19 in the hospitals. Media was crowded with information about the shutdown of elective medical help, and this possibly led to a false assumption that hospitals did not provide services unless it was COVID-19 infection. It is also speculated that positive effects of COVID-19 related lockdown such as reduction of air pollution or stress, increased sleep duration, reduced noise level, physical activity, and smoking possibly played a role in a lower incidence of acute coronary syndromes [6,7,12,13,14]. However, these hypotheses require more evidence. Many countries have also reported a reduction of hospitalizations for acute myocardial infarction: from −8.3% to −51.4% for STEMI and from −35% to −65.4% for NSTEMI. All these studies analyzed the period of the first COVID-19 pandemic wave, starting from the beginning of February and ending at the end of May 2020. Despite methodological differences (regarding time periods and cohorts) all studies confirmed clear tendency of the reduction of myocardial infarction cases [2,3,4,5,6,7,8,9,10,11,15,16,17,18,19]. Similarly, the reports have described greatly reduced number of percutaneous coronary interventions or cath-lab activations, reduction variating from −16% to −53% [5,6,12,20,21,22]. The decrease of hospitalizations due to AMI was observed not only in countries with high incidence of COVID-19, but also among nations with very low COVID-19 burden. There were on average 33 new daily cases of COVID-19 infection per million inhabitants reported during the spring lockdown in Lithuania [23]. This number is relatively small compared to many other countries during the first wave, but preventive actions taken by the government were like in Italy with high COVID-19 infection burden. Reduced coronary interventions and cath-lab activations could also be explained by increased priority to fibrinolysis due to new treatment algorithms during pandemic setting [21,24]. However, the rate of intravenous thrombolysis for STEMI patients did not increase during the lockdown in our center and was almost identical to the same period in 2019 (11.7% in 2019 and 11.9% in 2020, *p* = 1.0).

In our one-center sample, we did not find any statistically significant difference in the primary endpoint–all-cause mortality during hospital stay and 3 months follow up. However, in-hospital mortality of STEMI patients was higher in prepandemic era (9.4%) compared to 2020 (3.4%) group and this difference has narrowly missed statistical significance (*p* = 0.059). This finding could be attributed to the increased prevalence of cardiogenic shock among STEMI patients in 2019 (13.5% vs. 5.5% in 2020). It could be speculated that this difference is coincidental or that the fewer sickest patients decided to seek for help during pandemic. Some studies also compared mortality rates for acute coronary syndromes and demonstrated mixed results: while the majority of studies did not find a statistically significant increase of death rate, some investigations showed an increase of 7-day mortality variating from 1% to 3% [5,8,9,22,24]. Though these findings coincided with reports of prolonged ischemia times, it seems that pandemic situation did not disrupt the quality of primary PCI service on a grand scale. At the same time some studies reported growing numbers of out-of-hospital cardiac arrests by 56–58% [9,25], which raise concern of MI patients keeping away from the emergency departments. The studies from heavily affected areas reported increased mortality: in Italy, STEMI case fatality rate went up from 4.1% (2019) to 13.7% (2020) while NSTEMI case fatality rate was 1–3% during the pandemic, compared to 1–7% in 2019 [2]; in Hubei province of China, mortality rate for STEMI increased from 4.6% in 2019 to 7.3% in 2020 [11]. Wilson et al. reported that left ventricular ejection fraction of STEMI patients was significantly lower in the COVID-19 era (43.7% vs. 47.4%; *p* = 0.02). This could mean that despite not seeing any significant rise in mortality during the hospital stay and short follow-up afterwards, it still might lead to elevated morbidity and mortality in the future [5].

Second important finding of our study was that ischemia time intervals were prolonged for FMC-to-PCI and door-to-PCI times. While other ischemia time intervals did not differ significantly, the tendency of increase remained, which finally accumulated in total FMC-PCI time increase of 14 min to a total of 115 min. Moreover, almost half of STEMI patients surpassed recommended 120 min FMC-PCI time limit in 2020, while in 2019 this proportion was 25.8%. This is worrying finding since during the spring lockdown almost half of patients reached the threshold where fibrinolysis is favored compared to PCI according to the current European Society of Cardiology (ESC) guidelines [26]. On the other hand, FMC-PCI time was mostly prolonged by the increase of door to PCI time, and this increase was a mere 10 min—probably a justified amount having in mind additional safety measures, which had to be taken before bringing the patient into the cath-lab. Again, Lithuanian health system was not overloaded during the spring lockdown and ischemia times would probably were even longer if there were more COVID-19 patients, requiring medical help. Delays in PPCI service depends on the organization of emergency medical services and in-hospital PPCI pathways, which differs among countries and hospitals and might were reorganized in the setting of COVID-19. There are five PPCI centers in Lithuania located in five different cities and patients with STEMI are brought to the closest one. Reorganizing PPCI service (i.e., allocating one or more centers for COVID-19 positive or suspected patients) would inevitably prolong ischemia time and this option to our knowledge was not considered. Studies from different countries addressed delays in PPCI service: Kwok et al., reported increased average time in STEMI from symptom onset to hospital (150 vs. 135 min) and increased average door-to-balloon time (48 vs. 37 min) [24]; Abdelaziz et al. found an expansion of average time from symptom onset to FMC (227 min vs. 119 min) in STEMI patients (20). Italian study reported delays from symptom onset to coronary angiography by 39.2%, FMC-PCI time by 31.5% [2]. Tumminello et al. conducted a study, where they compared two in-hospital pathways for COVID-19 positive or suspected patients and low probability or no evidence of COVID-19 patients. They did not find a statistically significant difference in myocardial ischemia time between the two pathways, however, their sample size was relatively small [27]. The ESC guidance on the management of patients with STEMI during COVID-19 pandemic recognizes delays in the primary PCI pathways up to 60 min and recommends fibrinolysis if the target time cannot be achieved [28].

The limited availability of outpatient services and the fear of coming to the hospital during the lockdown could result in more STEMI patients presenting in poor clinical condition. However, the number of patients in cardiogenic shock or Killip III, IV class was not increased in our center. In fact, quite the opposite situation was observed. More STEMI patients were diagnosed with cardiogenic shock in 2019 compared to that of 2020 (13.5% vs. 5.5%, *p* = 0.043, respectively), while there was no significant difference in Killip class among NSTEMI patients. The reason for this finding remains unclear.

## 6. Limitations

We have collected data from only one PCI center in Lithuania, however, this center is one of the largest in the region. The follow-up is limited to 3 months, and thus could be too short to fully appreciate the impact of pandemic on STEMI patients’ survival. The availability of ambulance cards (the source of myocardial ischemia times) was limited in our relatively small sample size. Also, we could not collect the values of LVEF on discharge or during follow up.

## 7. Conclusions

Our data demonstrate that during the first COVID-19-related lockdown in a low-burden country, despite a decline in hospitalizations due to acute myocardial infarction and prolongation of myocardial ischemia time, the mortality of patients who underwent primary PCI during the hospital stay and 3-month follow-up did not increase. We also did not observe more complications after myocardial infarction. The quality of primary PCI service seems to be sustained in such a low-burden country as Lithuania.

## Figures and Tables

**Table 1:** Baseline characteristics of patients admitted with STEMI and NSTEMI.

Characteristic	STEMI Patients			NSTEMI Patients		
	2019 (*n* = 171)	2020 (*n* = 146)	*p*	2019 (*n* = 155)	2020 (*n* = 132)	*p*
Age	67.0 [59.0–76.5]	65.0 [57.0–74.0]	0.01	68.0 [59.0–78.0]	68.5 [59.0–76.0]	0.71
Female sex	48 (28.1)	34 (23.3)	0.40	57 (36.8)	46 (34.8)	0.83
Previous MI	26 (15.2)	18 (12.3)	0.57	37 (23.9)	34 (25.8)	0.82
Previous PCI	19 (11.1)	18 (12.3)	0.57	24 (15.5)	29 (22.0)	0.42
Previous CABG	2 (1.2)	1 (0.7)		3 (1.9)	3 (2.3)	
Arterial Hypertension	126 (73.7)	123 (84.8)	0.02	141 (91.0)	120 (90.9)	1.00
Dyslipidemia	136 (80.5)	130 (89.7)	0.04	134 (86.5)	115 (87.1)	1.00
Diabetes mellitus (Type 2)	23 (13.5)	21 (14.4)	0.27	26 (16.8)	37 (28.0)	0.04
Smoking	50 (56.2)	48 (76.2)	0.02	36 (63.2)	22 (45.8)	0.11
Chronic kidney disease (eGFR < 60 ml/min/1.73m^2^)	42 (24.7)	26 (17.9)	0.19	32 (20.6)	32 (24.2)	0.56

Values are median (25th–75th %) and number (percentage). MI–myocardial infarction; PCI–percutaneous coronary intervention; CABG–coronary artery bypass graft surgery; eGFR–estimated glomerular filtration rate.

**Table 2:** Acute coronary syndrome related characteristics of patients admitted with STEMI and NSTEMI.

Characteristic	STEMI Patients			NSTEMI Patients		
	2019 (*n* = 171)	2020 (*n* = 146)	*p*	2019 (*n* = 155)	2020 (*n* = 132)	*p*
MI localization						
Anterior	71 (41.5)	72 (49.3)	0.37	37 (48.7)	27 (50.0)	0.39
Inferior	91 (53.2)	68 (46.6)		15 (19.7)	15 (27.8)	
Other	9 (5.3)	6 (4.1)		24 (31.6)	12 (22.2)	
Killip class						
I	103 (60.2)	99 (67.8)	0.04	131 (84.5)	103 (78.0)	0.13
II	41 (24.0)	31 (21.2)		15 (9.7)	24 (18.2)	
III	4 (2.3)	8 (5.5)		6 (3.9)	2 (1.5)	
IV	23 (13.5)	8 (5.5)		3 (1.9)	3 (2.3)	
Out-of-hospital cardiac arrest	7 (4.1)	5 (3.4)	0.99	2 (1.3)	2 (1.5)	1.00
Peak hs-TnI, ng/l	22,448 [5339–64,349]	22,133 [4405–97,113]	0.69	1681 [376–9828]	1938 [499–6763]	0.82
LV EF, %	40 [35–47]	40 [35–47]	0.87	50 [45–55]	50 [40–55]	0.32
Length of hospital stay, days	8 [5–22]	7 [5–15]	0.58	6 [3–9]	6 [3–8]	0.96
Referral to cardiac rehab	112 (72.3)	80 (56.7)	0.008	83 (57.6)	46 (35.9)	0.001

Values are median (25th–75th %) and number (percentage). MI—myocardial infarction; hs-TnI—high sensitivity troponin I, LV EF–left ventricle ejection fraction.

**Table 3:** Coronary angiography findings and procedural characteristics.

Characteristic	STEMI Patients			NSTEMI Patients		
	2019 (*n* = 171)	2020 (*n* = 146)	*p*	2019 (*n* = 155)	2020 (*n* = 132)	*p*
No. of diseased coronary arteries						
1	59 (34.5)	50 (34.2)	0.72	27 (17.4)	32 (24.2)	0.17
2	52 (30.4)	50 (34.2)		67 (43.2)	44 (33.3)	
3	60 (35.1)	46 (31.5)		61 (39.4)	56 (42.4)	
Total occlusion of coronary artery	119 (69.6)	94 (64.4)	0.39	31 (20.0)	30 (22.7)	0.68
Culprit artery						
LAD	68 (39.8)	69 (47.3)	0.62	55 (38.2)	53 (41.7)	0.65
LCX	33 (19.3)	24 (16.4)		49 (34.0)	37 (29.1)	
RCA	67 (39.2)	51 (34.9)		29 (20.1)	28 (22.0)	
LM	2 (1.2)	2 (1.4)		2 (1.4)	4 (3.1)	
Graft	1 (0.6)	0 (0.0)		9 (6.2)	5 (3.9)	
TIMI flow pre-PCI						
0	115 (67.3)	91 (62.8)	0.78	24 (15.5)	22 (17.2)	0.93
1	9 (5.3)	11 (7.6)		7 (4.5)	6 (4.7)	
2	15 (8.8)	13 (9.0)		19 (12.3)	18 (14.1)	
3	32 (18.7)	30 (20.7)		105 (67.7)	82 (64.1)	
TIMI flow post-PCI						
0	3 (1.8)	2 (1.4)	0.98	1 (0.6)	1 (0.8)	0.56
1	2 (1.2)	2 (1.4)		0 (0.0)	1 (0.8)	
2	8 (4.7)	8 (5.5)		1 (0.6)	0 (0.0)	
3	158 (92.4)	133 (91.7)		153 (98.7)	126 (98.4)	
Conservative treatment	0 (0.0)	1 (0.7)	0.94	0 (0.0)	5 (3.8)	0.03
Complications related to PCI						
Distal embolisation	5 (2.9)	1 (0.7)	0.10			
Coronary artery dissection	0 (0.0)	1 (0.7)				
Coronary artery perforation	1 (0.6)	0 (0.0)				
Side branch occlusion	4 (2.3)	0 (0.0)				
Without complications	161 (94.2)	142 (98.6)		155 (100.0)	128 (99.2)	0.93
Revascularisation status after PCI						
Full revascularisation achieved	46 (27.2)	51 (35.2)	0.13	25 (16.1)	23 (17.8)	0.09
Second procedure planned for other lesions	27 (16.0)	28 (19.3)		15 (9.7)	4 (3.1)	
Conservative treatment for other lesions	96 (56.8)	66 (45.5)		115 (74.2)	102 (79.1)	
IABP or ECMO	7 (4.1)	4 (2.7)	0.73	2 (1.3)	0 (0.0)	0.55
Staged revascularisation during index hospital stay	14 (8.2)	17 (11.7)	0.39	6 (3.9)	4 (3.1)	0.96

Values are number (%). LAD—left anterior descending artery; LCX—left circumflex artery; RCA—right coronary artery; L—left main; TIMI—thrombolysis in myocardial infarction; PCI—percutaneous coronary intervention; IABP—intra-aortic balloon pump; ECMO—extracorporeal membrane oxygenation.

**Table 4:** Adverse events of patients admitted with STEMI and NSTEMI during hospital stay, and 3-month follow up.

Adverse Event	STEMI Patients			NSTEMI Patients		
	2019 (*n* = 171)	2020 (*n* = 146)	*p*	2019 (*n* = 155)	2020 (*n* = 132)	*p*
Worsening of heart failure during hospital stay	19 (11.1)	11 (7.5)	0.37	4 (2.6)	4 (3.0)	1.00
Stroke during hospital stay	0 (0.0)	0 (0.0)	NA	0 (0.0)	0 (0.0)	NA
Recurrent MI during hospital stay	0 (0.0)	0 (0.0)	NA	0 (0.0)	0 (0.0)	NA
In-hospital death	15 (9.4)	5 (3.4)	0.059	2 (1.3)	1 (0.8)	1.00
Death at 3 months follow-up	18 (10.5)	10 (6.8)	0.34	4 (2.6)	7 (5.3)	0.37

Values are number (%). MI—myocardial infarction; worsening of heart failure was defined as deterioration of clinical condition requiring treatment in intensive cardiac care unit.

## Data Availability

The data presented in this study are available on request from the corresponding author.
